# Deviations in the gut microbiota of neonates affected by maternal group B Streptococcus colonization

**DOI:** 10.1186/s12866-021-02204-3

**Published:** 2021-05-05

**Authors:** Yue-feng Li, Xue-lei Gong, Su-xiang Chen, Kejian Wang, Yan-hua Jiang

**Affiliations:** 1 Department of Pediatrics, Shenzhen Luohu Maternity and Child Health Hospital, Shenzhen, 518019 China; 2 The Third Affiliated Hospital of Shandong First Medical University Affiliated Hospital of Shandong Academy of Medical Sciences, Jinan, 250031 China; 3 Departments of Obstetrics and Gynecology, Shenzhen Luohu Maternity and Child Health Hospital, No.2013, Taibai Road, Luohu District, Shenzhen, 518019 China

**Keywords:** Group B Streptococcus (GBS) colonization, Gut microbiota, Microarray-based technique

## Abstract

**Background:**

Group B Streptococcus (GBS) infection is the leading cause of septicemia, meningitis, and pneumonia in neonates. Aberrant gut colonization in early life may predispose children to various diseases in adulthood. However, the associations between gut microbial changes and GBS colonization is still unclear.

**Results:**

The composition and diversity of meconium microbiota in GBS group were similar to that of healthy controls. However, we identified several specific taxa that were differentially abundant between the two groups (linear discriminant analysis (LDA) effect size (LEfSe): *p* < 0.05, LDA > 2.0). Particularly, the relative abundance of *Lactobacillus paracasei* was significantly reduced, indicating a role in GBS colonization.

**Conclusions:**

Our study presented a series of bacterial species colonized by GBS, thus providing novel evidence in support of initial intestinal microbiota dysbiosis in the neonates with mother’s GBS colonization.

**Supplementary Information:**

The online version contains supplementary material available at 10.1186/s12866-021-02204-3.

## Background

Group B Streptococcus (GBS) are β-hemolytic and Gram-positive bacteria, which are recognized as a leading cause of neonatal early-onset sepsis (EOS), meningitis, and pneumonia [1, 2]. The mother-to-child vertical transmission is the major GBS infection route in neonatal periods. Previous studies have shown that the prevalence of GBS colonization in vagina during pregnancy is approximately 10–30% [3, 4] and the neonatal morbidity rate for acquiring GBS through birth canal is 60% [5]. The implementation of intrapartum antibiotic prophylaxis (IAP) in pregnant women with GBS colonization is a preventive treatment for reducing the risk of GBS-induced neonatal EOS [6]. However, IAP might also disrupt the balance between microbial members of the gut microbiota [7–9].

Recent evidences indicated that the disturbance of gut microbiome has been involved in potential prenatal and early life of infant [10, 11]. For example, Cassidy-Bushrow et al. observed that *Clostridiaceae*, *Ruminococcoceae*, and *Enterococcaceae* were significantly enriched in infants of GBS positive (GBS+) mothers compared to infants of GBS negative (GBS-) ones [12]. Rosen et al. reported 18 taxa that were found to be significantly associated with GBS carriage [13].

Meconium, as the first stool of infant, is made up of materials ingested in utero and considered as a good source for studying the microbiome of the maternal-fetal interface [14]. Based on the advancement in the technologies and methodologies to identify the microbiota, a number of studies have highlighted the possible microbial presence in the meconium which is partially similar to adult gut microbiome [15] and the microbiome of placenta/amniotic fluid [16]. These studies support the colonization of the fetal gut that may begin in utero due to contact with the placenta and/or amniotic fluid.

Although these aforementioned studies provided clues about the origin of microbiome of meconium and how GBS alter the vaginal microbiome of pregnant women, the origin of prenatal microbiome, expecially the placental microbiome, is still a lively debated focus in the recent year [17]. A study by de Goffau MC and his colleagues showed that human placenta has no microbiome except for potential pathogens such as GBS colonization [18]. Regarding the relationship between the gut microbiota of infants and maternal GBS colonization remains also largely unknown. In this study, we adopted a new microarray-based technique [19] to characterize the fecal samples of the neonates in GBS+ group as compared with control group. The study aimed to investigate the influence of maternal GBS colonization on the gut microbiome of newborns, with the intention of improving perinatal infant care.

## Results

### The clinical information in the study

**Fig. 1:**
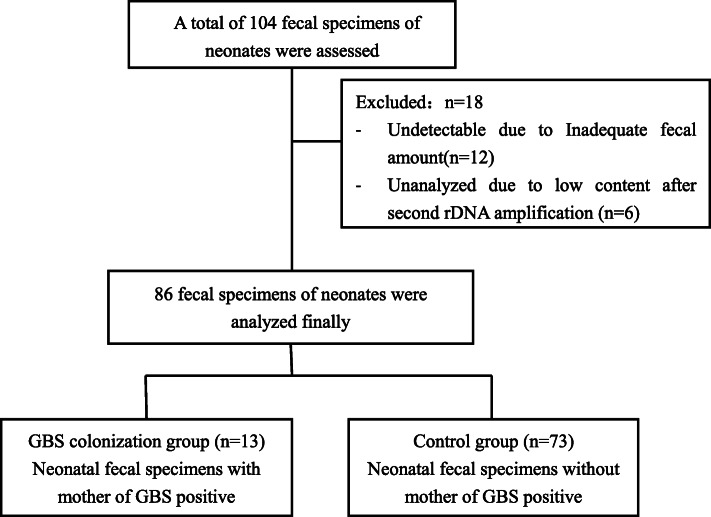
The flowchart of this study

A total of 104 neonatal fecal specimens were collected during the study period. Of these, 12 fecal samples were undetectable due to inadequate amount of total DNA after extraction and 6 fecal samples were further excluded due to low content of 16S rDNA after amplification. Finally, 86 fecal samples from neonates were analyzed. The flowchart of the study is shown in Fig. 1.

The clinical characteristics of the 86 neonates are shown in Table 1. There were no difference between two groups except in the term of gestational age and antibiotics exposure after birth.

**Table 1:** The clinical information between GBS+ and control groups

Parameters	GBS + group (*n* = 13)	Control group (*n* = 73)	OR (95%CI)	*P* value
Neonatal features				
Gestational age, W	39.5 ± 0.6	38.8 ± 1.9	–	0.009
Birth weight, g	3192 ± 218	3121 ± 515	–	0.4
Male, (n, %)	8 (61.5)	39 (53.4)	1.4 (0.4 ~ 4.7)	0.6
Cesarean section (n, %)	2 (15.4)	30 (41.1)	0.3 (0.05 ~ 1.3)	0.1
Antibiotics exposure	10 (76.9)	30 (41.1)	4.8 (1.2 ~ 18.8)	0.017
Mother complication				
PROM (n, %)	2 (15.4)	17 (23.3)	0.6 (0.1 ~ 2.9)	0.8
Intrapartum fever history	0 (0)	7 (9.6)	0.8 (0.8 ~ 0.9)	0.5
GDM (n, %)	1 (7.7)	27 (37.0)	0.1 (0.02 ~ 1.15)	0.07
Placental abruption	0 (0)	1 (1.4)	–	1.0^#^
MSAF (n, %)	1 (7.7)	23 (31.5)	0.2 (0.02 ~ 1.44)	0.1
IAP exposure	11 (84.6)	44 (60.3)	3.6 (0.7 ~ 17.6)	0.17

*PROM* Prelabor rupture of the membranes, *GDM* Gestational diabetes mellitus, *MSAF* Meconium- stained amniotic fluid, *IPA* Intrapartum antibiotics prophylaxis

^#^Fisher exact test

### Comparison of α- and β-diversity between two groups

**Fig. 2:**
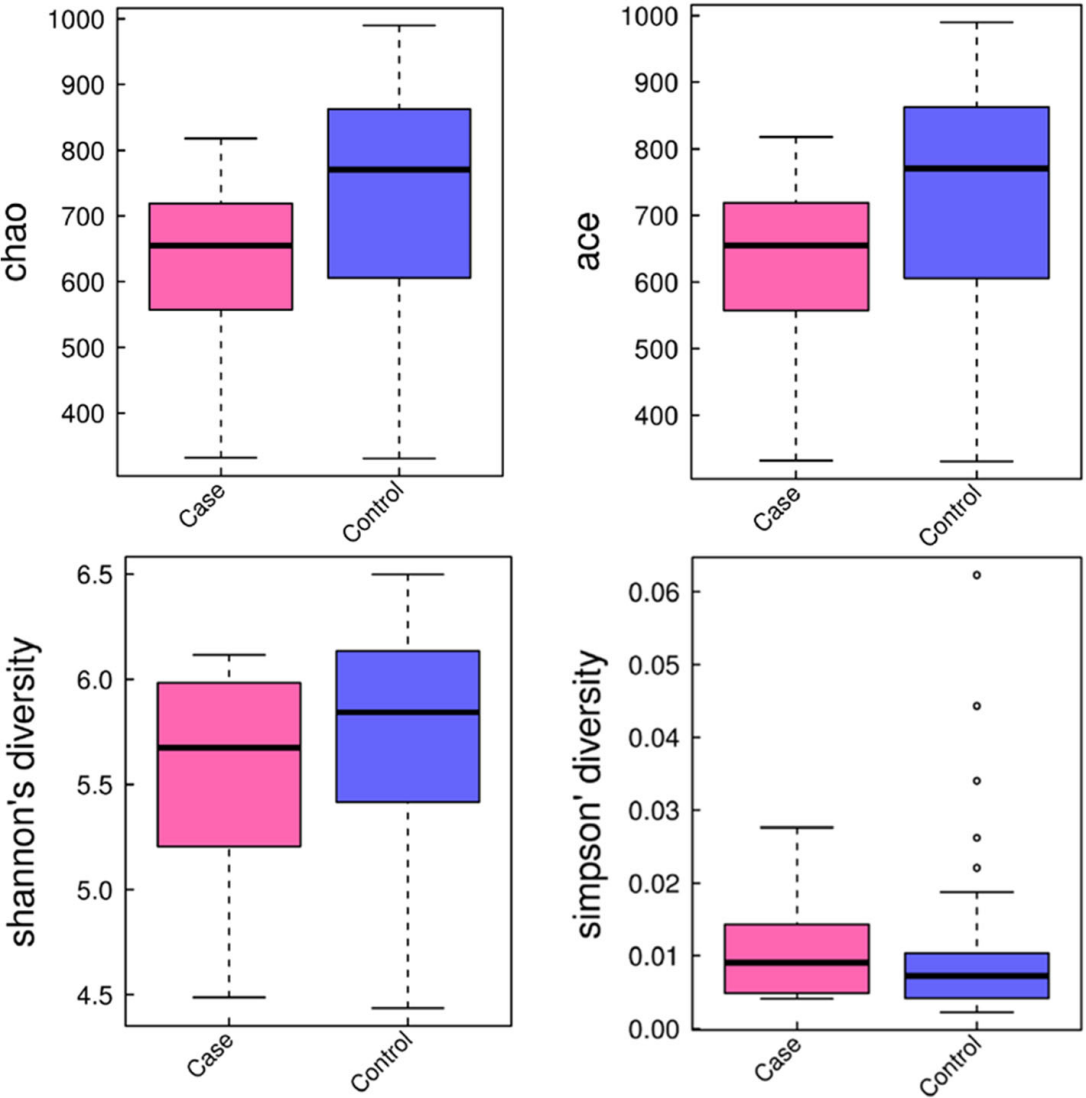
Box plot of Chao, Ace, Shannon and Simpson indexes

**Fig. 3:**
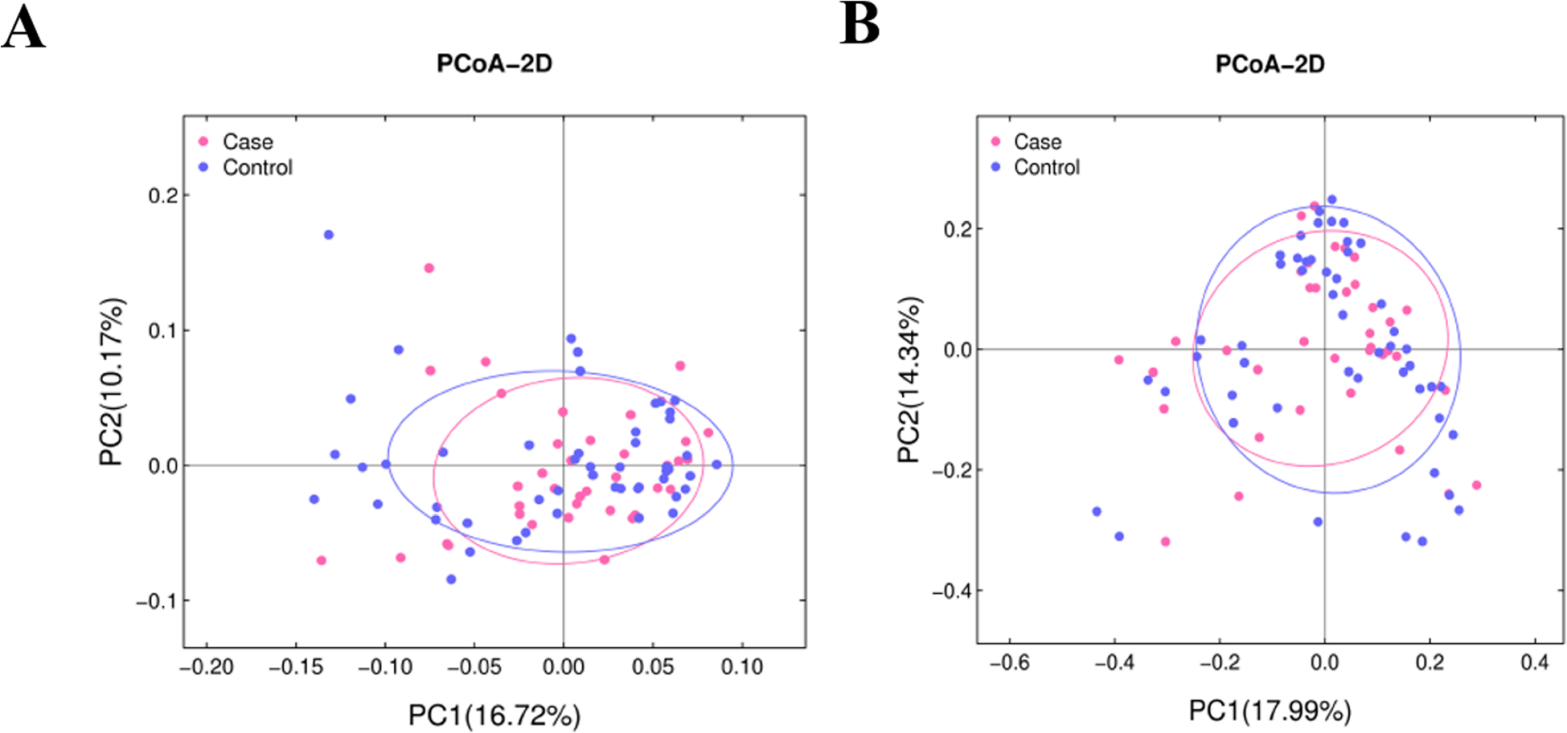
Gut bacterial community analysis of infants in GBS+ and control groups. Principal coordinates analysis (PCoA) plots based on weighted Unifrac distance (**a**) and Bray Curtis distance (**b**)

To evaluate the differences in composition of gut microbiota, we performed α- and β-diversity analyses. Several α-diversity indexes including Chao, Ace, Shannon and Simpson (Fig. 2) indicated no significant difference in species richness and diversity between GBS+ and control groups. As to β-diversity, as β-diversity indicators, were applied to estimate the dissimilarity between samples. PCoA plots based on weighted Unifrac distance and Bray Curtis distance showed that the controls clustered more tightly than the infants in GBS+ group (Fig. 3), indicating similar bacterial compositions in the controls. Furthermore, the exposure to infants to antibiotics did not significantly change the gut microbiota in infants of GBS infected group and control group (Figure S1).

### Alteration of taxa in the GBS+ and control groups

**Fig. 4:**
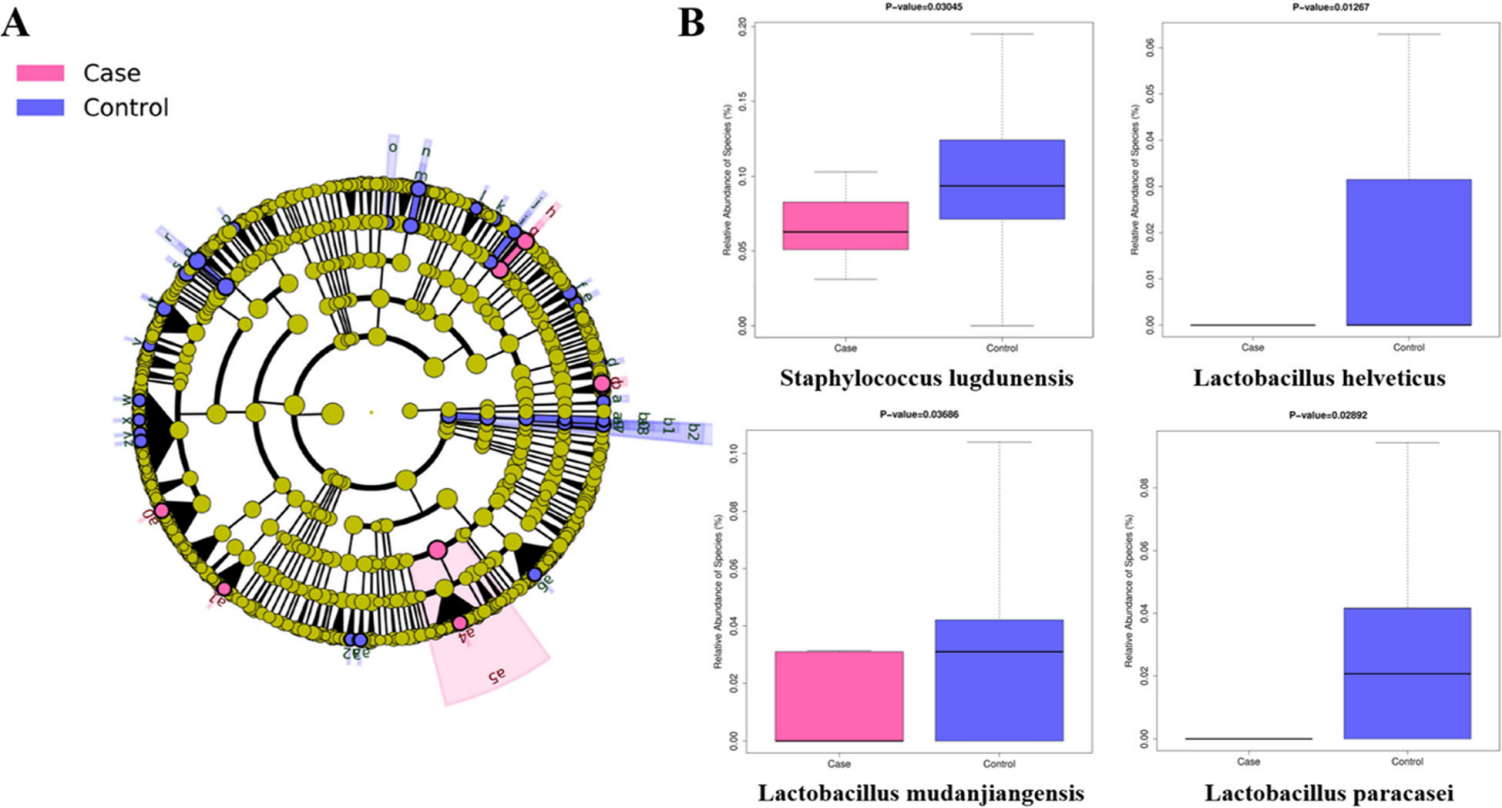
Different profiles of gut microbiota in meconium between infants in GBS+ and control groups. **a** Cladogram of differentially abundant taxa, from the phylum level down to the species level. **b** The relative abundance of certain taxa associated with GBS infection

To identify the specific taxa associated with GBS infection, a comparison of the microbiota between the infants in GBS+ and control groups was conducted by the Linear discriminant (LDA) and effect size (LEfSe) approach. A cladogram represented the significant structure of the gut microbiota from phylum level to species level (Fig. 4a), which listed a collection of the differential abundant bacteria between two groups. Particularly, the abundance of *Staphylococcus lugdunensis*, *Lactobacillus helveticus*, *Lactobacillus mudanjiangensis*, *Lactobacillus paracasei* in the infants with GBS + group were reduced as compared to the controls.

## Discussion

The imbalance of bacterial communities in infants has a profound impact on host’s health, but there is insufficient evidence to suggest the associations between dysbiosis in meconium and bacterial colonization such as GBS colonization. In this context, identification of the factors affecting the morbidity for gestational GBS-related colonization is an important issue that needs to be addressed.

In this study, bacterial composition and diversity showed no significant differences between infants in GBS+ and control groups. However, we found a lower abundance of *Staphylococcus* and *Lactobacillus* in the infants with GBS+ mothers, which was in line with previous studies. *Staphylococcus lugdunensis* is a coagulase-negative *Staphylococcus* [20], which has been implicated as the main pathogen in various infections, including central nervous system infections, urinary tract infections, and systemic infections [20–23]. A study from Japan reported that the GBS detection was correlated with significantly lower probability of coagulase-negative *Staphylococcus* [24]. Furthermore, Altoparlak et al. reported that the decreased level of *Lactobacillus* species was associated with detection of GBS colonization [25]. Kubota et al. demonstrated that GBS positive women had lower percentages of *Lactobacillus* than GBS negative women [24]. It should be noted that certain *Lactobacillus* such as *Lactobacillus paracasei* had the capabilities to prevent GBS adherence to vaginal epithelial cells [26], and antimicrobial activity of *Lactobacillus* against GBS had been documented in vitro [27]. Moreover, this lower *Lactobacillus* species had been detected in the neonatal EOS recently [28]. Thus, the reduced abundance of above genera might limit the protective role of microbiome so as to increase susceptibility to infection.

Although intrapartum antibiotics prophylaxis (IAP) is the most effective measurement to reduce the risk of GBS-induced neonatal EOS [6], it can impact neonatal gut microbiota [29] and until the first 3 months after birth, thus increasing the prevalence of antibiotic resistance genes [30]. In our study, infants exposed to antibiotics after birth were significant higher in GBS group (76.9%) than control group (41.1%) due to GBS colonization (Table 1), but meconium microbiota of infants exposed or unexposed to antibiotics were no difference in GBS infected group and control group (Figure S1). Hence, this imbalance of bacterial communities within 24 h of life in our study might be closely correlated with mother’s GBS colonization in vaginal tract, which was in line with previous studies [12].

There were also certain limitations of our results. First, this study was conducted in a single center with a relatively small sample set. Second, potential influence of nutrition intakes during pregnancy was not taken into consideration. Third, the confound influence of antibiotic exposure on meconium microbiota prior to labor or operative period for C-section and immediately after birth couldn’t address completely due to the shortage of study design and relatively small sample size. Furthermore, we do not have access to the matched maternal microbiome samples, thus evidence tracing the origin of meconium microbiome is required in further study.

## Conclusion

In summary, our findings add to a growing body of knowledge about the association between GBS colonization and neonatal meconium. Our results demonstrated the potential features of gut microbiota in neonatal early life born to mother with GBS colonization, which may lead to new biomarkers and innovative therapeutic approaches for perinatal infant care.

## Methods

### Study design and sample collection

The Ethics Committee of Shenzhen Luohu Maternity and Child Health Hospital has approved all of the research procedures. Under the procedure approved by the Institutional Review Board (registry number: LL201804007), informed consent was given by the parents of the newborns.

The high-risk neonates admitted to the department of neonatology from May, 2018 to Jul, 2019 were enrolled after receiving informed consent from the parents. Preterm infants with extreme asphyxia (stage III), fetal chromosomal abnormalities, cyanotic congenital cardiac failure, congenital intestinal atresia, gastroschisis, omphalocele, excessive upper gastric intestinal bleeding, or parental permission deficiency/refusal were excluded from the study.

The fecal samples were collected in 30 ~ 50 g from a sterilized diaper by using the sterile container having an equal volume of sterile cryoprotectant within 24 h after birth and transported immediately to lab on ice and stored at − 80 °C for further studies. The specialized senior nurses were responsible for this work and the small sample spoon could not touch other neonatal body sites when collecting.

The GBS culture from mother’s vaginal swab were conducted at 36 week gestational age for term labor or prior to delivery for premature labor. The clinical information, treatment and lab data of mothers and neonates were extracted from medical records.

### DNA extraction and labeling

Bacterial DNA from the stool samples was processed following a previously published protocol [19]. In brief, DNA was collected using the Stool DNA Extraction Kit (Halgen, Ltd., Zhongshan, China) and amplified in a PCR with standardized primers that covered the 16S rRNA gene V1-V9 regions. The PCR products were explicitly labeled without purification for array hybridization.

### Microarray hybridization

Previous protocols were followed to perform microarray hybridization [19]. In general, Cy5- and Cy3-labeled sample DNA were combined and loaded into a hybridization tank. After 3.5 h incubation, the slides were manually washed and automatically screened using a dual-channel (Genepix 4000B) scanner to calculation the mean signal strength of Cy5/Cy3 ratio, by which the relative abundance of each bacterial species is given.

### Data analysis

Alpha-diversity was measured using default parameters and QIIME tools (version 1.9.0, http://qiime.org/) [31]. Wilcoxon rank-sum test was used to measure the disparities in alpha-diversities between classes. Analyzes of principal coordinates analysis (PCoA) and non-metric multidimensional scaling (NMDS) were performed using QIIME modules and visualized with the “ggplot2” package of R software (version 3.5.2). PERMANOVA test determines whether groups of samples are significantly different from one another using the ADONIS permutation-based statistical test in ‘Vegan’ package of R software. Linear Discriminant Analysis (LDA) Impact Size (LEfSe) tool [32] was adopted to analyze the disparity between classes of bacterial organisms. A threshold of > 2.0 was set for the logarithmic LDA score in order to take into account discriminant features [32].

## Supplementary Information

**Additional file 1.**

## Data Availability

The datasets supporting the conclusions of this article are included within the article and additional files. All these data are available from the corresponding author on reasonable request.
