# Supplementary material for: Deviations in the gut microbiota of neonates affected by maternal group B Streptococcus colonization
Source: BMC Microbiol. 2021 May 5;21:140. doi: 10.1186/s12866-021-02204-3 (PMC8097833; doi:10.1186/s12866-021-02204-3)
Supplement: Supplementary file 1 — Additional file 1. [file 12866_2021_2204_MOESM1_ESM.docx]

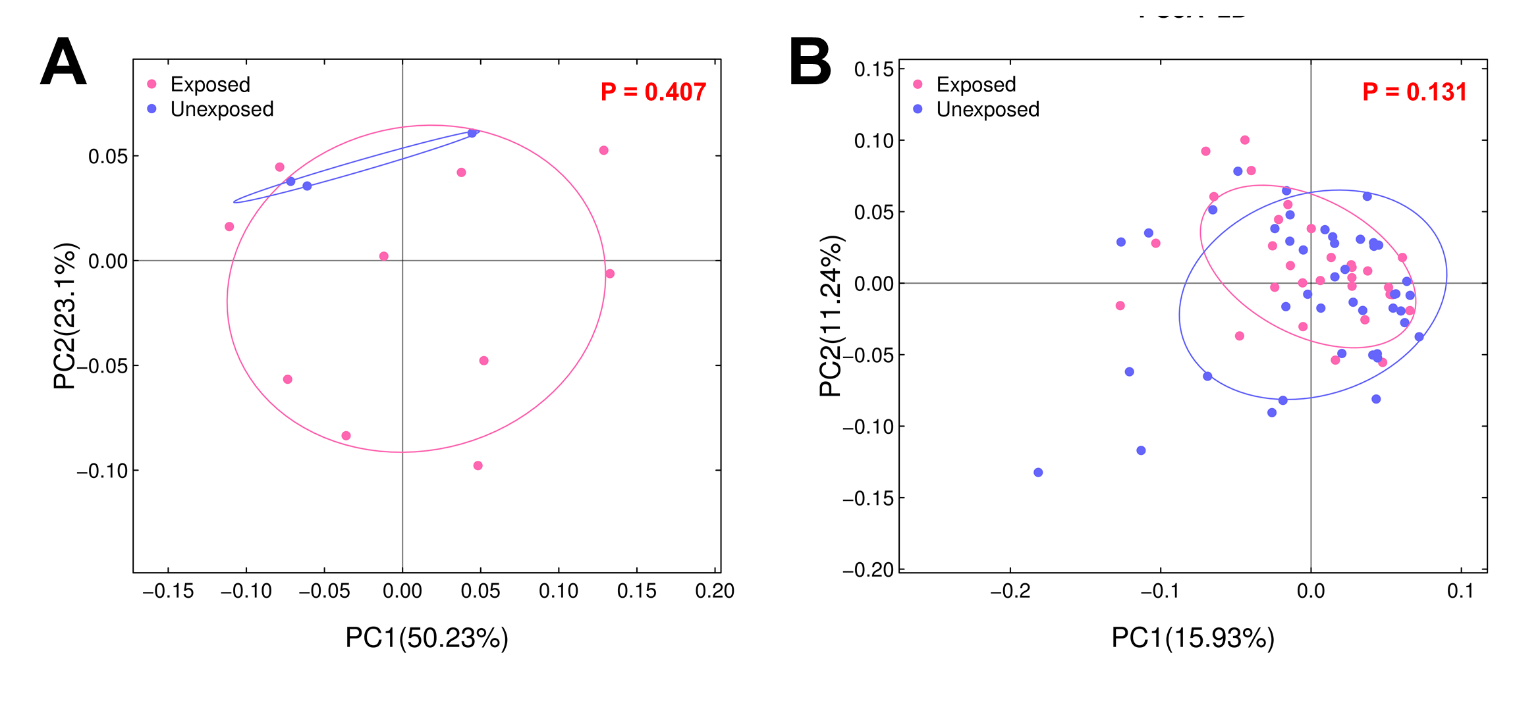


**Figure S1.** Principal coordinates analysis (PCoA) plots of infants exposed or unexposed to antibiotics in GBS infected group (A) and control group (B).
